# Understanding cellular proliferation activity in breast cancer using multi-compartment model of transverse relaxation time mapping on 3T MRI

**DOI:** 10.3389/fonc.2025.1482112

**Published:** 2025-01-30

**Authors:** Kangwa Alex Nkonde, Sai Man Cheung, Nicholas Senn, Jiabao He

**Affiliations:** ^1^ Translational and Clinical Research Institute, Faculty of Medical Sciences, Newcastle University, Newcastle upon Tyne, United Kingdom; ^2^ Department of Physics, School of Natural and Applied Sciences, Mulungushi University, Kabwe, Zambia; ^3^ Institute of Medical Sciences, School of Medicine, Medical Sciences and Nutrition, University of Aberdeen, Aberdeen, United Kingdom

**Keywords:** neoadjuvant treatment, Bayesian, Ki-67, intra-cellular, extra-cellular, biochemical environment

## Abstract

**Introduction:**

Precise understanding of proliferative activity in breast cancer holds significant value in the monitoring of neoadjuvant treatment, while current immunostaining of Ki-67 from biopsy or resected tumour suffers from partial sampling error. Multi-compartment model of transverse relaxation time has been proposed to differentiate intra- and extra-cellular space and biochemical environment but susceptible to noise, with recent development of Bayesian algorithm suggested to improve robustness. We hence hypothesise that intra- and extra-cellular transverse relaxation times using Bayesian algorithm might be sensitive to proliferative activity.

**Materials and methods:**

Twenty whole tumour specimens freshly excised from patients with invasive ductal carcinoma were scanned on a 3 T clinical scanner. The overall transverse relaxation time was computed using a single-compartment model with the non-linear least squares algorithm, while intra- and extra-cellular transverse relaxation times were computed using a multi-compartment model with the Bayesian algorithm. Immunostaining of Ki-67 was conducted, yielding 9 and 11 cases with high and low proliferating activities respectively.

**Results:**

For single-compartment model, there was a significant higher overall transverse relaxation time (*p* = 0.031) in high (83.55 ± 7.38 ms) against low (73.30 ± 11.30 ms) proliferating tumours. For multi-compartment model, there was a significant higher intra-cellular transverse relaxation time (*p* = 0.047) in high (73.52 ± 10.92 ms) against low (61.30 ± 14.01 ms) proliferating tumours. There was no significant difference in extra-cellular transverse relaxation time (*p* = 0.203) between high and low proliferating tumours.

**Conclusions:**

Overall and Bayesian intra-cellular transverse relaxation times are associated with proliferative activities in breast tumours, potentially serving as a non-invasive imaging marker for neoadjuvant treatment monitoring.

## 1 Introduction

New pharmaceutical options and the improvement in screening services have improved the 5-year survival rate of breast cancer (1, 2), the most prevalent cancer worldwide (3), from 79.9% between 2000 – 2004 to 86.3% between 2014 – 2018 in the UK. Neoadjuvant therapies, often a combination of chemotherapy and hormonal therapy, are increasingly used to improve surgical outcomes by downstaging large tumours to facilitate breast conservation surgery (4). Neoadjuvant therapy is typically lengthy and costly, exposing non-responding patients to unnecessary potential adverse effects (5, 6). Rapid tumour growth, a central prognostic indicator of breast cancer, can be estimated using the proliferative activity marker Ki-67, highlighting nuclear protein expressed during cell division (7). However, Ki-67 using biopsy is invasive with narrow spatial coverage, suboptimal for accurately estimating intrinsically heterogeneous proliferative activity across the whole tumour during treatment (8, 9). Hence, an imaging marker of proliferative activity is central to effective neoadjuvant treatment monitoring in breast cancer.

PET tracer of 3’-deoxy-3’-[18F] fluorothymidine (FLT) has been shown to correlate with Ki-67 in breast cancer (10), but causes myelosuppression, peripheral neuropathy and nausea at high doses (7). Peri-tumoural texture features of kurtosis, skewness and entropy, based on maximum enhancement from dynamic contrast-enhanced (DCE) MRI are associated with proliferative activity in large cohorts (11, 12), but the primary sensitivity of DCE MRI towards angiogenesis compromises specificity (13). Apparent diffusion coefficient from diffusion weighted imaging (DWI) can distinguish between low and high proliferating breast tumours (11, 14), but is susceptible to biological noise (15). Both peri-tumoural lipid composition from chemical shift-encoded imaging (CSEI) (16) and perfusion fraction from intravoxel incoherent motion (IVIM) MRI (17) have shown correlations against proliferative activity, but the primary sensitivity to tissue composition and perfusion limits specificity (18). Hence, an imaging approach specific to proliferative activity with low susceptibility to biological noise is highly desirable.

Transverse relaxation time mapping has been shown to highlight tumour pathology (19), but only offers a crude picture of tissue microenvironment (20). Rapidly proliferating tumours are associated with elevated angiogenesis to support accelerated metabolic activities (21) exacerbating vascular permeability and fluid efflux (22), and the consequent increased free water pool and a diluted biochemical microenvironment (21, 23) lead to elevated transverse relaxation time in high proliferating breast tumours as observed *in vivo* (24). Intra-cellular transverse relaxation time, although more susceptible to noise compared to overall transverse relaxation time, is more specific to alterations in intra-cellular processes (25), potentially revealing tumour response to chemotherapy targeting at rapid cell division at an early stage (26). Recent application of multi-compartment model differentiates lumen, intra- and extra-cellular spaces, as demonstrated in luminal water imaging in prostate cancer (27), classification of adipocytic tumour in soft tissue (28), differentiation of layers in articular cartilage (29) and estimation of myelin volume in the brain (30). Although multi-compartment model has been suggested as a marker of proliferative activity in breast cancer (31), the susceptibility to erroneous attribution of noise (20, 29) significantly curtailed the clinical application. Bayesian algorithm, using probabilistic constraints between neighbouring image voxels, was subsequently developed to reduce susceptibility to noise (32–34), and we have recently demonstrated the value in improving imaging methods for neoadjuvant treatment monitoring (17). We hence hypothesise that multi-compartment model of transverse relaxation time mapping using Bayesian algorithm might be a sensitive marker of proliferative activity.

## 2 Materials and methods

**Figure 1:**
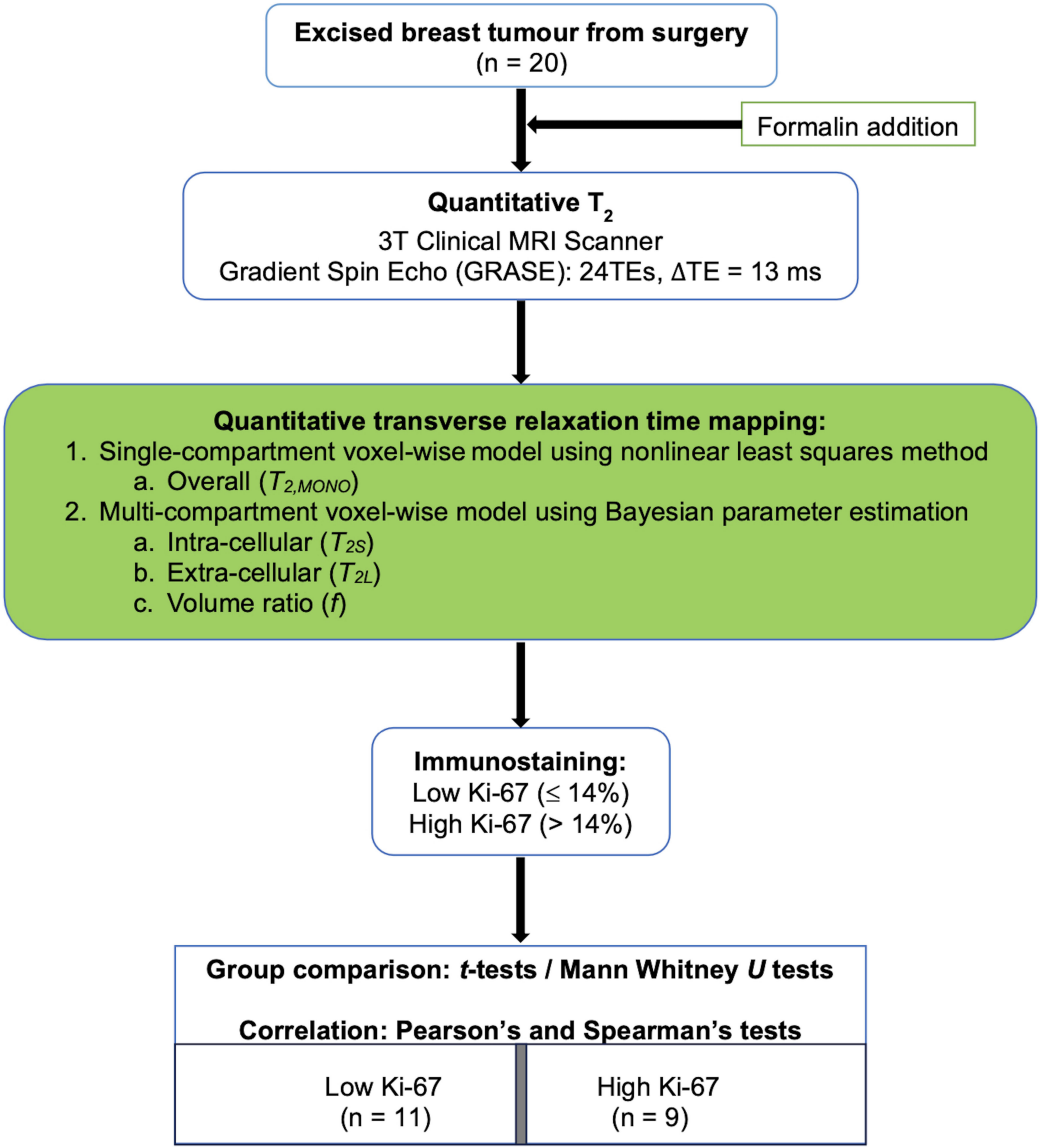
Study design. Twenty specimens freshly excised from patients with invasive ductal carcinoma grades II and III were imaged on a clinical 3T MRI scanner using quantitative transverse relaxation time mapping, with subsequent histopathological confirmation of low and high cellular proliferation activity. The overall transverse relaxation time was computed voxel-wise using a single-compartment model, while the intra- and extra-cellular transverse relaxation times and volume ratio were calculated voxel-wise using a multi-compartment model.

To probe this hypothesis, a cross-sectional study was conducted on 20 breast tumour specimens freshly excised from patients. Multi-compartment model of transverse relaxation time maps using Bayesian algorithm was performed to derive the intra- and extra-cellular transverse relaxation times and the volume ratio of each breast tumour specimen (**Figure 1**). The study was approved by the North-West – Greater Manchester East Research Ethics Committee (REC Reference: 16/NW/0221), and signed written informed consent was obtained from all participants prior to the study.

### 2.1 Clinical procedure

Fresh tumour specimens were excised from twenty female patients (age mean 57 years, range 35 to 78 years) with invasive ductal carcinoma (IDC) (10 grade II and 10 grade III). The study included patients with tumour size larger than 1.5 cm in diameter on mammography. Patients with previous malignancies and undergoing chemotherapy or radiotherapy prior to surgery were excluded from the study. Upon excision, the tumour specimens were immobilised in a 10% buffered formalin solution in a sealed container before imaging. Routine histopathological examination was performed to determine the histological tumour diameter, grade and Nottingham Prognostic Index (NPI), with oestrogen receptor (ER), progesterone receptor (PR) and human epidermal growth factor receptor 2 (HER2) (35). Tumour cellular proliferation activity marker Ki-67 (36) was assessed semi-quantitatively after single-batch immunostaining, with high Ki-67 indicating that more than 14% (37) of tumour cell nuclei staining positive above the background (38, 39), and there were 9 and 11 cases with high and low proliferative activities respectively.

### 2.2 Transverse relaxation time mapping

Quantitative transverse relaxation time images were acquired from each specimen on a clinical 3T MRI scanner (Achieva TX, Philips Healthcare, Netherlands) using a 32-channel receiver coil for signal detection and the body coil for uniform transmission. The images were acquired using a multishot gradient and spin echo (GRASE) sequence (40), with 24 echoes, initial echo time of 13 ms, echo spacing of 13 ms, repetition time of 9943 ms, field of view of 141 × 141 mm^2^, slice thickness of 2.2 mm, and image resolution of 2.2 × 2.2 mm^2^. The overall transverse relaxation time was computed voxel-wise using single-compartment model with the non-linear least squares method in MATLAB (R2021a, MathWorks Inc., Natick, MA, USA). The intra- and extra-cellular transverse relaxation times and volume ratio were calculated voxel-wise using the multi-compartment model with the Bayesian algorithm (33). The whole tumour was delineated for each specimen using MRIcron (v1.0.20190902, University of South Carolina, Colombia, USA) on conventional DWI images acquired at *b* value at 800 smm^-2^ using pulsed gradient spin echo (PGSE) sequence, with the necrotic regions excluded from the analysis. The overall transverse relaxation time, intra- and extra-cellular transverse relaxation times, and intra- and extra-cellular volumes were computed as the mean within the whole tumour from corresponding quantitative maps for each specimen, and the volume ratio subsequently calculated as the ratio between intra-cellular volume against combined intra- and extra-cellular volumes (27).

### 2.3 Statistical analysis

All statistical analysis was performed in the SPSS software 27.0 (IBM Corp, Armonk, NY, USA). Shapiro-Wilk test for normality was performed on all the outcome measures. Independent sample *t*-tests or Mann-Whitney *U* tests were conducted to compare the overall, intra- and extra-cellular transverse relaxation times and volume ratio between tumours with high and low proliferative activities. Pearson’s correlation tests were performed between the overall transverse relaxation time, intra-cellular transverse relaxation time and volume ratio against the tumour diameter, with Spearman’s rank correlation test performed between the extra-cellular transverse relaxation time against the tumour diameter. Spearman’s rank correlation tests were performed between the transverse relaxation times and volume ratio against NPI. A *p*-value < 0.05 was considered statistically significant.

## 3 Results

The patient demography and statistical findings are shown in **Tables 1**, **2** respectively. There were no significant differences in patient characteristics between the high and low Ki-67 groups.

**Table 1:** Patient demography.

Demographic	All (n = 20)	Ki-67 expression		*p-value*
		Low (n = 11)	High (n = 9)	
Age (years)^^^	58 ± 14	63 ± 11	51 ± 12	0.052
Tumour Size (mm)^^^	24.9 ± 5.7	24.1 ± 6.6	25.9 ± 4.7	0.489
Nottingham Prognostic Index (NPI) ^†^	4.44 (3.50 – 4.59)	3.70 (3.50 – 4.57)	4.44 (4.42 – 4.60)	0.199
Histological grade^*^				
II	10	8	2	0.070
III	10	3	7	
Lymphovascular Invasion (LVI)^*^				
LVI Positive	12	7	5	1
LVI Negative	8	4	4	
Necrosis^*^				
Present	9	6	3	0.406
Absent	11	5	6	
Tumour Infiltrating Lymphocytes (TILs)^*^				
Low	13	7	6	1
Intermediate	4	3	1	0.591
High	3	1	2	0.566

^^^Independent samples *t*-test for continuous variables; ^†^Mann-Whitney *U*-test for continuous non-parametric variables; ^*^Fisher's exact test for categorical variables.

Breast cancer patients with high and low proliferative activity marker Ki-67 expression are shown for each group and the entire cohort. Values are expressed as mean and standard deviation for normally distributed data, and median and interquartile range for non-normally distributed data. Histopathological entries are expressed as the number of positive observations.

**Table 2:** Relaxometry properties in breast cancer.

Parameter	Molecular subtypes				Ki-67 expression			Tumour size		NPI	
	*Lum A* *(n = 9)*	*Lum B-HER2 (*–) *(n = 4)*	*Lum B-HER2(+)* *(n = 4)*	*TN* *(n = 3)*	*Low* *(n = 11)*	*High* *(n = 9)*	*p-value*	*r/ρ*	*p-value*	*ρ*	*p-value*
*f* (%)	43.84 ± 9.86	33.77 ± 5.68	36.82 ± 10.87	29.01 ± 7.17	41.65 ± 10.08	33.64 ± 8.33	0.073	0.02^^^	0.922	-0.17^+^	0.473
T_2, MONO_ (ms)	71.19 ± 11.45	84.88 ± 8.41	79.76 ± 5.71	84.63 ± 4.54	73.30 ± 11.30	83.55 ± 7.38	0.031*	0.22^^^	0.360	-0.06^+^	0.806
T_2L_ (ms)	156.85 ± 21.08	144.74 ± 9.05	154.62 ± 5.98	149.79 ± 11.23	156.56 ± 19.16	147.38 ± 8.84	0.203	-0.50^+^	0.025*	0.02^+^	0.947
T_2S_ (ms)	59.01 ± 14.56	74.91 ± 11.35	66.00 ± 4.66	77.43 ± 9.58	61.30 ± 14.01	73.52 ± 10.92	0.047*	-0.01^^^	0.979	-0.05^+^	0.828

HER2, Human epidermal growth factor receptor 2; Lum, Luminal; TN, Triple negative. ^^^Pearson’s correlation coefficient (*r*); *^+^* Spearman’s rank correlation (*ρ)*.

The overall transverse relaxation time (T_2, MONO_), intra- and extra-cellular transverse relaxation times (T_2S_, T_2L_) and volume ratio (*f*) between molecular subtypes, and low and high Ki-67 expression are shown. The correlations of T_2, MONO_, T_2S_, T_2L_ and *f* against tumour diameter and Nottingham Prognostic Index (NPI) are also shown. Statistically significant differences (p < 0.05) are in bold and marked by '*'.

**Figure 2:**
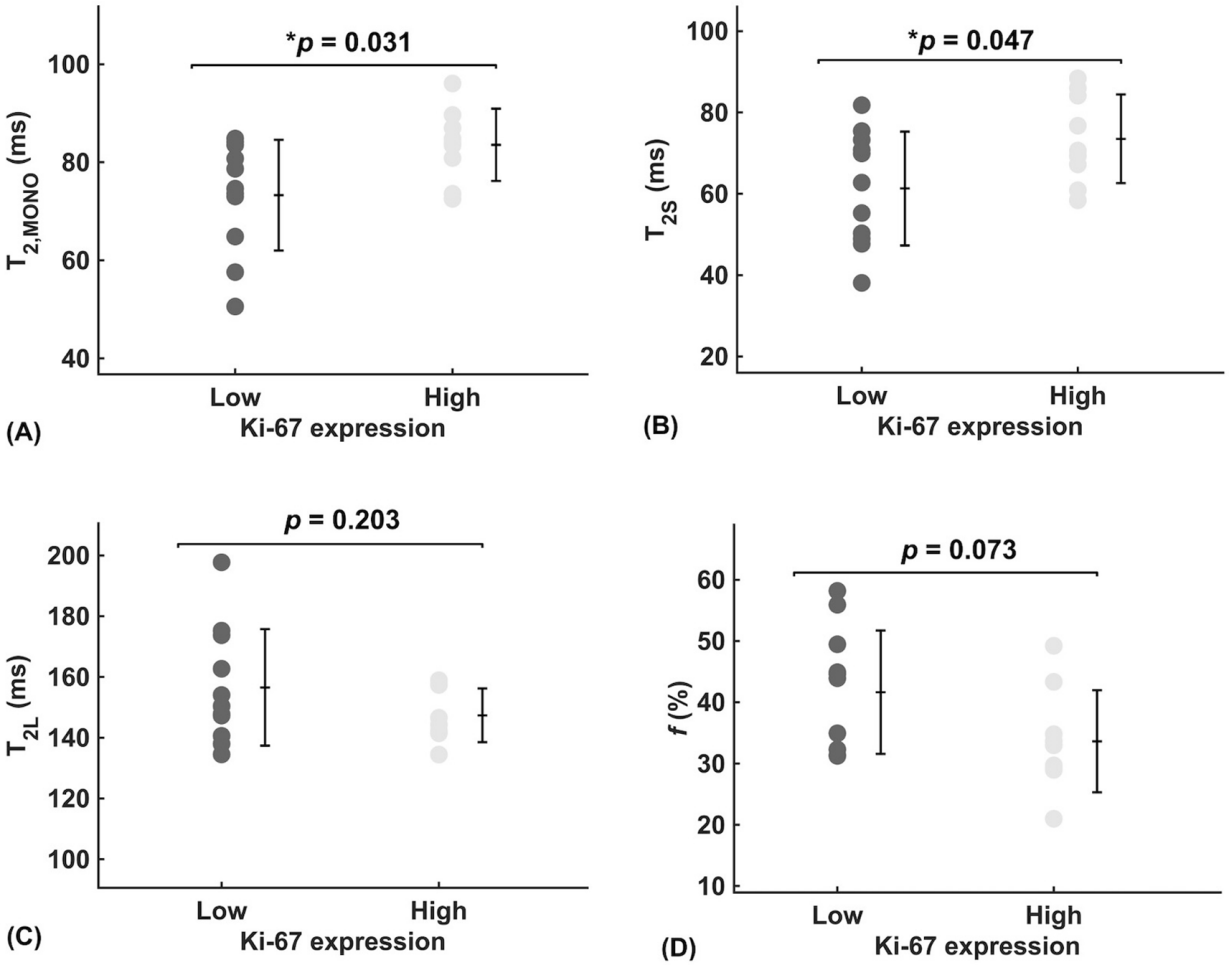
Group comparison of relaxometry properties between low and high Ki-67 expression group in breast cancer (n = 11, 9). **(A)** Overall transverse relaxation times (T_2, MONO_) are significantly higher in high proliferating tumours than in low proliferating tumours, potentially indicating a diluted biochemical environment. **(B)** Intra-cellular transverse relaxation times (T_2S_) are significantly higher in high proliferating tumours than in low proliferating tumours, potentially indicating a dilution of the intra-cellular biochemical environment. **(C)** There was no significant difference in extra-cellular transverse relaxation times (T_2L_) between high and low proliferating tumours, suggesting biochemical homeostasis. **(D)** The near-significantly higher volume ratio (*f*), in low proliferating tumours than in high proliferating tumours, suggests possible physical homeostasis between intra- and extra-cellular fluid volumes. Each dot represents the mean ROI of the parameter, arranged by low (Ki-67 ≤ 14%) and high (Ki-67 > 14%) proliferative activity. Error bars represent the mean ± standard deviation. Two-tailed independent *t*-tests were conducted, with *p*-values provided; statistically significant results (*p* < 0.05) are marked by ‘*’.

There was a significant higher overall transverse relaxation time (*p* = 0.031, **Figure 2A**) in high Ki-67 tumours (83.55 ± 7.38 ms) against low Ki-67 tumours (73.30 ± 11.30 ms). There was a significant higher intra-cellular transverse relaxation time (*p* = 0.047, **Figure 2B**) in high Ki-67 tumours (73.52 ± 10.92 ms) against low Ki-67 tumours (61.30 ± 14.01 ms). There was no significant difference (*p* = 0.203, **Figure 2C**) in the extra-cellular transverse relaxation time between high Ki-67 tumours (147.38 ± 8.84 ms) and low Ki-67 tumours (156.56 ± 19.16 ms). There was no significant difference (*p* = 0.073, **Figure 2D**) in volume ratio between high Ki-67 tumours (33.64 ± 8.33%) and low Ki-67 tumours (41.65 ± 10.08%).

**Figure 3:**
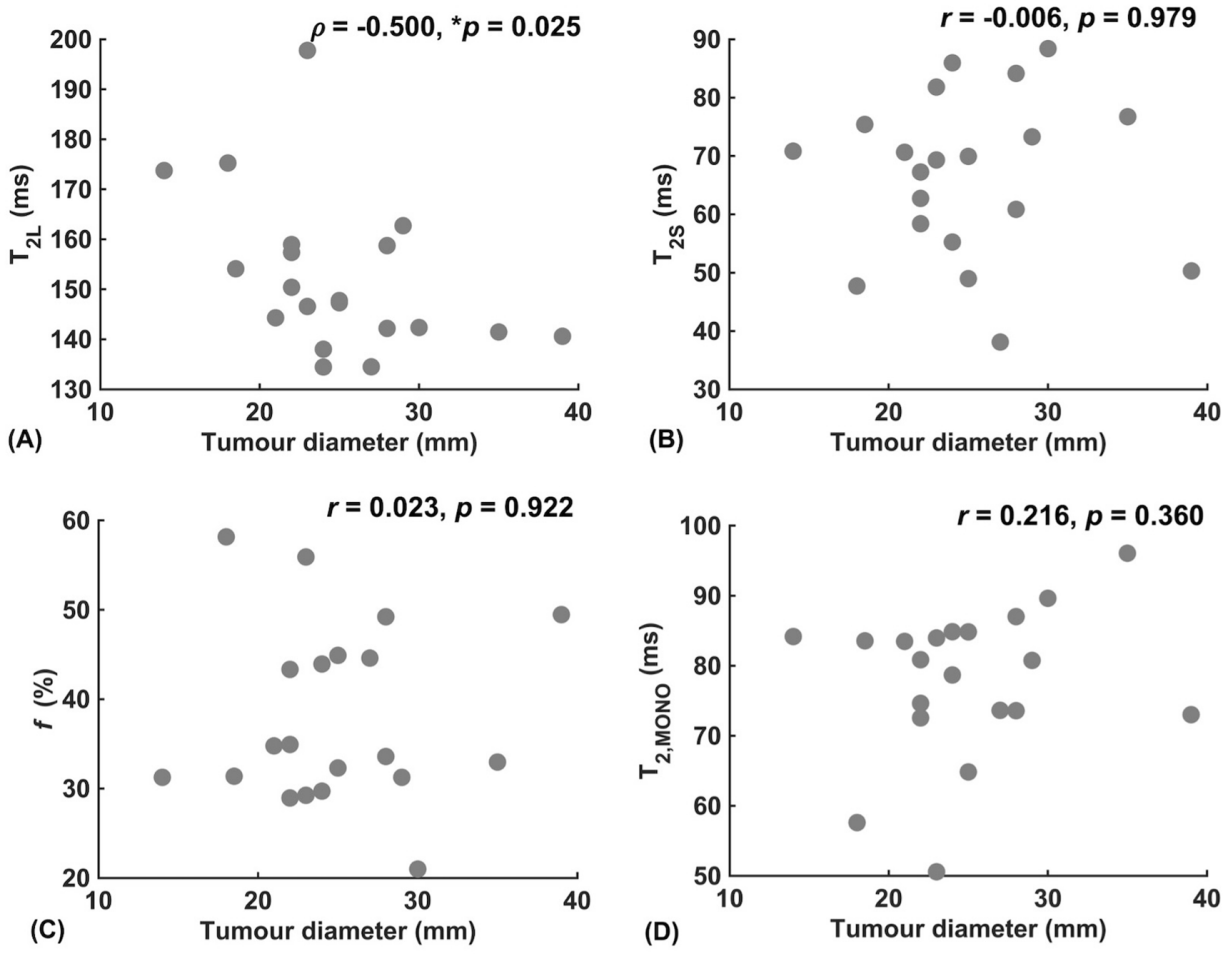
Correlation analysis between relaxometry properties and breast tumour diameter (n = 20). **(A)** Extra-cellular transverse relaxation time (T_2L_). **(B)** Intra-cellular transverse relaxation time (T_2S_). **(C)** Volume ratio (*f*). **(D)** Overall transverse relaxation time (T_2, MONO_). Statistically significant correlations (*p* < 0.05) are marked by ‘*’.

**Figure 4:**
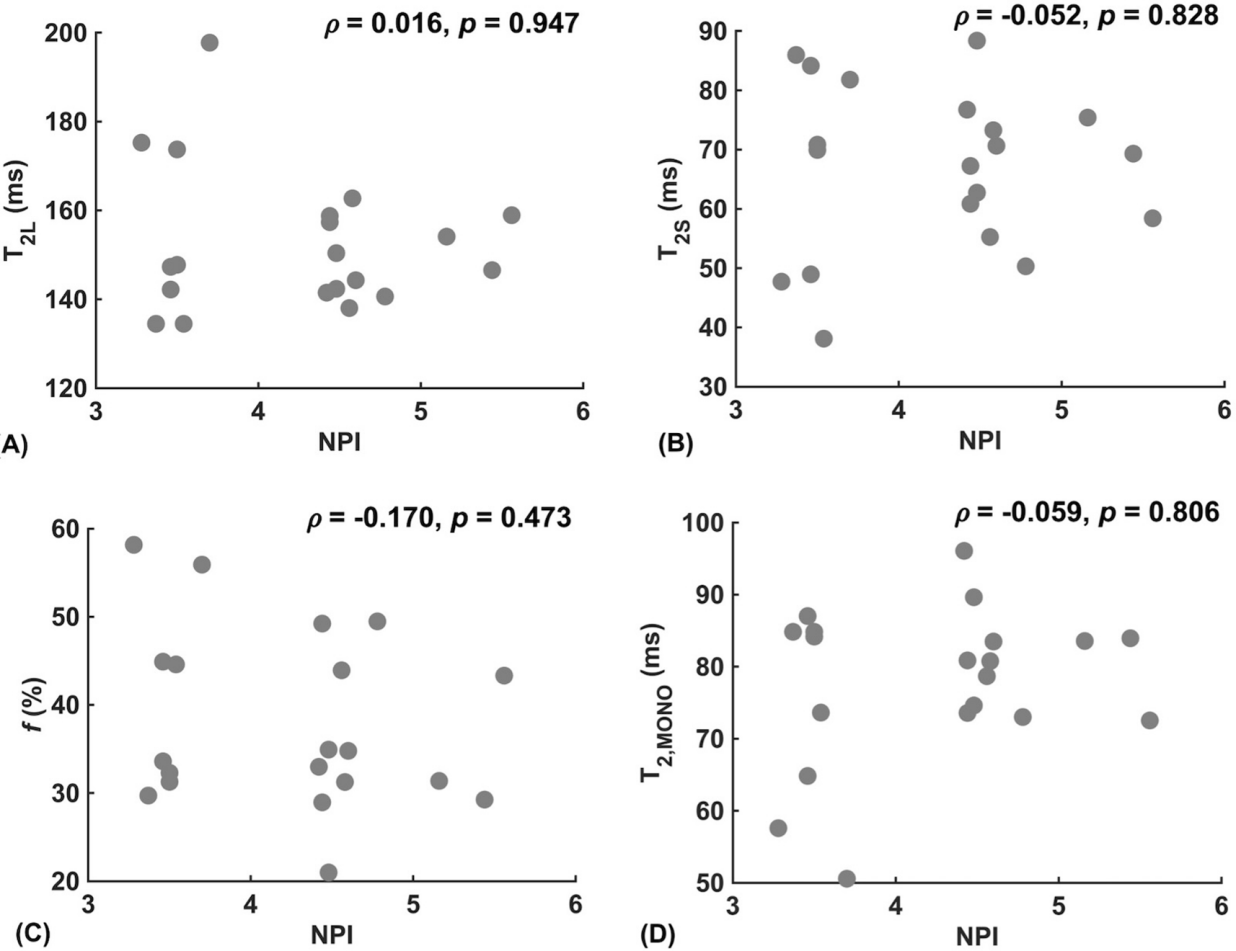
Correlation analysis between relaxometry properties and Nottingham Prognostic Index (NPI) in breast cancer (n = 20). **(A)** Extra-cellular transverse relaxation time (T_2L_). **(B)** Intra-cellular transverse relaxation time (T_2S_). **(C)** Volume ratio (*f*). **(D)** Overall transverse relaxation time (T_2, MONO_).

**Figure 5:**
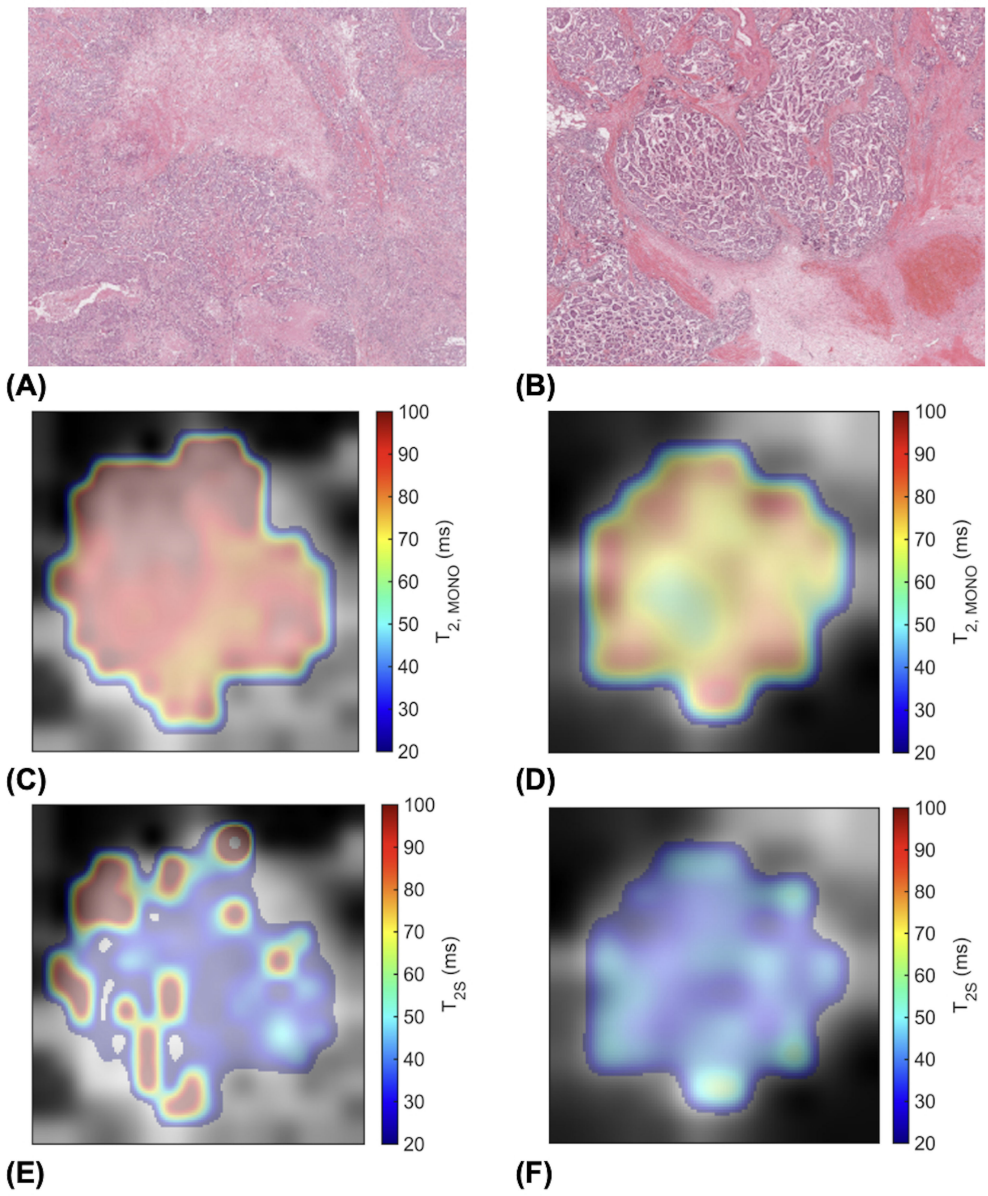
High (Ki-67 > 14%) and low (Ki-67 ≤ 14%) proliferating breast tumour specimens in haematoxylin and eosin (H & E) staining and the corresponding transverse relaxation time maps. Sections are taken from the greatest dimension of the tumour diameter. Magnification, x20. **(A)** A high Ki-67 expression of 49.18%. **(B)** A low Ki-67 expression of 4.96%. **(C)** Overall transverse relaxation time (T_2, MONO_) map of the high Ki-67 specimen. **(D)** T_2, MONO_ map of the low Ki-67 specimen. **(E)** Intra-cellular transverse relaxation time (T_2S_) map of the high Ki-67 specimen. **(F)** T_2S_ map of the low Ki-67 specimen.

Against tumour diameter, there was a significant negative correlation in extra-cellular transverse relaxation time (*ρ = -*0.50*, p =* 0.025, **Figure 3A**), but not in intra-cellular transverse relaxation time (*p =* 0.979), volume ratio (*p =* 0.922) and overall transverse relaxation time (*p =* 0.360) (**Figure 3**). There were no significant correlations between extra-cellular transverse relaxation time (*p =* 0.947), intra-cellular transverse relaxation time (*p =* 0.828), volume ratio (*p =* 0.473) and overall transverse relaxation time (*p =* 0.806) against NPI (**Figure 4**). Typical breast tumour specimens of high and low Ki-67 expression and the corresponding maps of overall and intra-cellular transverse relaxation times are shown in **Figure 5**, and maps of extra-cellular transverse relaxation time and volume ratio are shown in **Supplementary Figure S1** (**Supplementary Material 1**).

## 4 Discussion

In this study, we found significant differences between high and low proliferating tumours in overall and intra-cellular transverse relaxation times, but not in extra-cellular transverse relaxation time and volume ratio. We found a significant negative correlation of the extra-cellular transverse relaxation time against tumour diameter, but not in the remaining three relaxometry properties. No significant correlation of the four relaxometry properties against NPI was found.

The elevated overall transverse relaxation time in high proliferating tumours indicates a reduced rate of signal dissipation potentially due to a diluted biochemical environment (23). The rapid metabolic activity in high proliferating tumours results in increased free water pools in the tumour (21), while the enhanced angiogenesis exacerbates vascular permeability and fluid leakage into the tumour (22). Hence, the central characteristics of rapidly proliferating tumours (41), of increased metabolic activity and enhanced angiogenesis, contribute to the diluted biochemical environment in high proliferating tumours (42). The elevated intra-cellular transverse relaxation time in high proliferating tumours indicates a reduced rate of signal dissipation potentially due to a diluted intra-cellular biochemical environment (43). The rapid cell division in high proliferating tumours, as highlighted by Ki-67 (21), demands upregulated transportation of amino acids from the cytoplasmic space to sustain biosynthesis in the nucleus (44), leading to a diluted intra-cellular biochemical environment. The stronger statistical significance in overall transverse relaxation time compared to intra-cellular transverse relaxation time might be the result of lower intra-cellular signal as a fraction of the overall signal, but might also indicate the presence of feedforward and feedback pathways. Angiogenesis exacerbates vascular permeability to support accelerated metabolic activity, compounding fluid efflux in both the intra- and extra-cellular compartments (22), potentially leading to a more significant elevation in the overall transverse relaxation time in high proliferating tumours (23). In contrast, the elevated transportation of amino acids into the nucleus from the cytoplasmic space of the intra-cellular compartment to support accelerated biosynthesis in rapidly proliferating tumours partially cancels out the fluid efflux, potentially leading to a more subdued increase in the intra-cellular transverse relaxation time (44). Chemotherapies, targeting at rapid cell division, significantly alter the intra-cellular biochemical environment (26), hence sensitive imaging markers might contribute to early response monitoring (25). The lack of significant difference in the extra-cellular transverse relaxation time indicates an undisturbed rate of signal dissipation potentially reflecting biochemical homeostasis, although unnaturalised free radicals as by-products from aerobic glycolysis (45) are actively expelled to extra-cellular volume (46), promoting fibrosis (47), angiogenesis (22) and reduction of free water (48). The lack of significant difference in the volume ratio reflects the physical homeostasis of intra- and extra-cellular fluid volumes (49) critical for minimal disruption of osmotic pressure and transportation across cellular compartments in tumours (21). Vesicles, carrying oxidative stress signal transmitters, are released in the extra-cellular volume, but are actively transported back into the intra-cellular volume to modulate oxidative stress and fluid imbalance (50). The near-significant difference in the volume ratio compared to the significant difference in overall and intra-cellular transverse relaxation times might be the result of intrinsic lower effect size compared to measurement error, but might potentially indicate the secondary effects of structural changes (51) following biochemical alteration (52). Protective homeostatic mechanism (49), modulating fluid exchange through vesicles between the compartments (50), may dampen the effects of proliferative activities on the volume ratio with the same trend but weaker significance against transverse relaxation times (53).

The negative correlation between extra-cellular transverse relaxation time and tumour diameter might be due to the increased cellularity and reduced extra-cellular free water pools associated with larger tumours (48). The significant negative correlation between extra-cellular transverse relaxation time and tumour size indicates a more concentrated biochemical environment or less free water at larger tumour size. Larger tumours suffer from hypoxia due to inadequate vascular supply (54) and extra-cellular matrix remodelling (55), and the increase in the macromolecules to water ratio in the extra-cellular compartment (56) lead to a reduction in extra-cellular transverse relaxation time (57). There was no significant correlation between overall transverse relaxation time, intra-cellular transverse relaxation time and volume ratio with tumour diameter, reflecting the insensitivity of the three relaxometry properties to morphological tumour size. The lack of significant correlation between overall transverse relaxation time, intra-cellular and extra-cellular transverse relaxation times and volume ratio against NPI indicated that the four relaxometry properties, although potential treatment monitoring markers, might not be sensitive to recurrence and metastatic risks revealed by NPI (58).

Although an understanding of the heterogeneity in the overall and intra-cellular transverse relaxation times required thorough quantitative analysis across the whole tumour using appropriate texture features, however conjectural visual exploration might serve to develop future hypothesis. Intra-cellular transverse relaxation time showed sparse focal elevation at the edges of high proliferating tumours but more homogeneity in low proliferating tumours, while overall transverse relaxation time showed more homogeneous elevation in high proliferating tumours but exhibited bands of elevation at the edges of low proliferating tumours (**Figure 5**). The focal increase in intra-cellular free water content, manifested as a lengthening of intra-cellular transverse relaxation time, in high proliferating tumours might reflect the elevated transportation of amino acids into the nucleus from the cytoplasmic space to sustain biosynthesis in the nucleus at the advancing edge of the tumour (44). The bands of increased free water content, manifested as the elevation of overall transverse relaxation time, at the edge of low proliferating tumours might reflect the presence of angiogenesis and oedema concentrated at the tumour margin (22, 42), in contrast to their widespread presence across the whole tumour in high proliferating tumours (24). Precise spatial correlation between imaging and pathology, demanding co-localisation at a fine cellular resolution, was not feasible during this study, and should be performed in future investigations.

To our knowledge, this is the first study investigating the relaxation properties as a marker of breast tumour proliferative activity using Bayesian algorithm in conjunction with multi-compartment model to mitigate the impact from noise. This study only recruited patients with large breast tumours to take into account the reliance of Bayesian algorithm on neighbouring voxels, and necrotic tumour cores were excluded from analysis to avoid confounding factors from non-viable tissue in large tumours. The tumours were freshly excised to eliminate biological noise and further improve analysis accuracy, and imaging was performed immediately to avoid the alteration to relaxation properties from formalin submersion (59). The cohort size was small due to inclusion criteria on large tumour to accommodate novel imaging methods, and future large cohort studies on patients are critical to fully understand the value of the imaging marker. Future simulation study on the variability and high resolution imaging from ultra-high field MRI can support the establishment of a confidence range in sensitivity and an estimation of the highest effective image resolution, allowing studies to be carried out at a higher image resolution and in smaller tumours.

## 5 Conclusions

Overall and Bayesian intra-cellular transverse relaxation times are associated with proliferative activities in breast tumours, potentially serving as a non-invasive imaging marker for neoadjuvant treatment monitoring.

## Data Availability

The raw data supporting the conclusions of this article will be made available by the authors, without undue reservation.
