# Supplementary material for: Understanding cellular proliferation activity in breast cancer using multi-compartment model of transverse relaxation time mapping on 3T MRI
Source: Front Oncol. 2025 Jan 30;15:1482112. doi: 10.3389/fonc.2025.1482112 (PMC11821498; doi:10.3389/fonc.2025.1482112)
Supplement: Supplementary file 1 [file DataSheet1.pdf]

Supplementary Material

Supplement 1: Additional figure for the clinical study

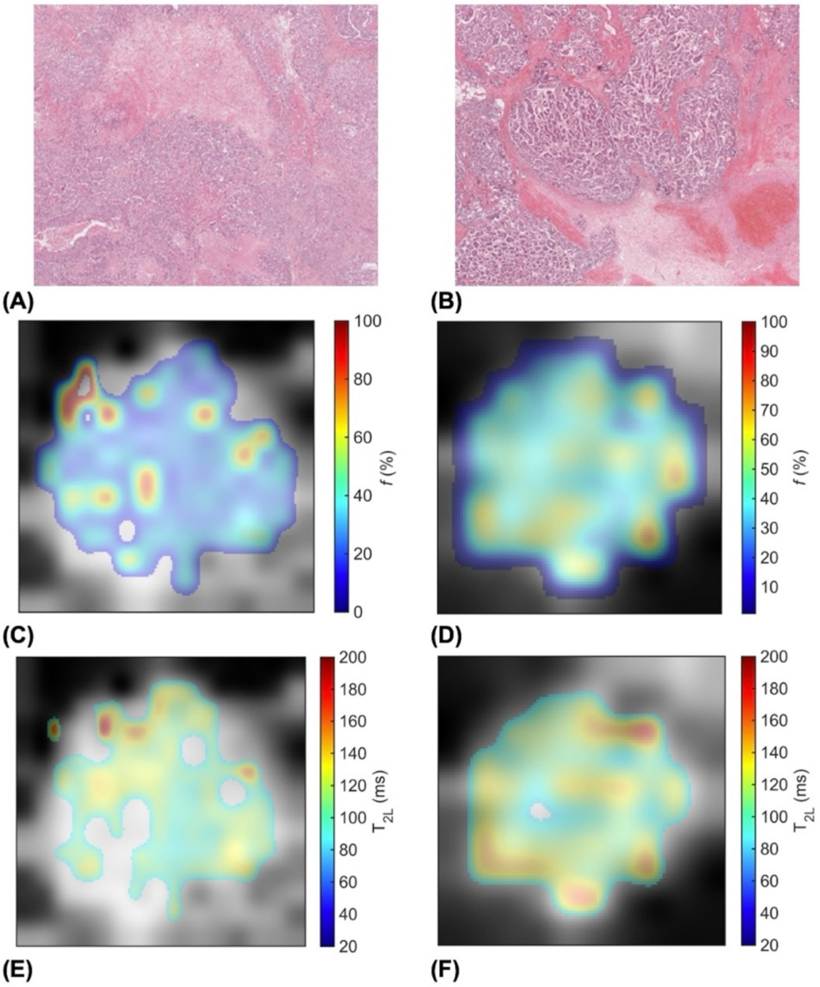

Figure S1. High (Ki-67 > 14%) and low (Ki-67 ≤ 14%) proliferating breast tumour specimens in haematoxylin and eosin (H & E) staining and the corresponding volume ratio and transverse relaxation time maps. Sections are taken from the greatest dimension of the tumour diameter. Magnification, x20. (A) A high Ki-67 expression of 49.18%. **(B)** A low Ki-67 expression of 4.96%. **(C)** Volume ratio (*f*) map of the high Ki-67 specimen. **(D)** *f* map of the low Ki-67 specimen. **(E)** Extra-cellular transverse relaxation time (T_{2L}) map of the high Ki-67 specimen. **(F).** T_{2L} map of the low Ki-67 specimen.
